# A DELPHI study on aspects of study design to overcome knowledge gaps on the burden of disease caused by serogroup B invasive meningococcal disease

**DOI:** 10.1186/s12955-019-1159-0

**Published:** 2019-05-22

**Authors:** Ole Marten, Florian Koerber, David Bloom, Monika Bullinger, Corinne Buysse, Hannah Christensen, Philippe De Wals, Christian Dohna-Schwake, Philipp Henneke, Markus Kirchner, Markus Knuf, Burkhard Lawrenz, Andrea L. Monteiro, Joseph Patrick Sevilla, Nicolas Van de Velde, Robert Welte, Claire Wright, Wolfgang Greiner

**Affiliations:** 1 Department of Health Economics and Health Care Management, School of Public Health, Bielefeld University, Universitätsstr. 25, 33615 Bielefeld, Germany; 2 GSK, Prinzregentenplatz 9, 81675 Munich, Germany; 3 Department of Global Health and Population, Harvard T.H. Chan School of Public Health, 665 Huntington Avenue, Boston, Massachusetts 02115 USA; 4 Department of Medical Psychology, University Medical Center Hamburg-Eppendorf, Martinistr. 52, 20246 Hamburg, Germany; 5 Department of Pediatrics, Intensive Care Unit, Erasmus Medical Center, Sophia Children’s Hospital, Wytemaweg 80, 3015 CN Rotterdam, The Netherlands; 6 Population Health Sciences, Bristol Medical School, University of Bristol, Oakfield House, Oakfield Grove, Bristol, BS8 2BN UK; 7 Department of Social and Preventive Medicine, Laval University, Avenue de la Médecine 1050, Quebec City, G1V 0A6 Canada; 8 Pediatrics I, University Hospital of Essen, Hufelandstr. 55, 45147 Essen, Germany; 9 Center for Pediatrics and Adolescent Medicine and Center for Chronic Immunodeficiency, Medical Center - University of Freiburg, 79106 Freiburg, Germany; 10 HELIOS Dr. Horst Schmidt Kliniken, Children‘s Hospital, Ludwig-Erhard-Str. 100, 65199 Wiesbaden, Germany; 11 Pediatric Infectious Diseases, University Medicine, Mainz, 55131 Germany; 12 Association of Professional Pediatricians and Adolescent Medicine Specialists in Germany (Berufsverband der Kinder- und Jugendärzte e.V., BVKJ), Mielenforster Str. 2, 51069 Köln, Germany; 13 Private Practice for Pediatrics and Adolescent Medicine, Grafenstr. 80, 59821 Arnsberg, Germany; 14 Asas Avaliacoes Economicas Em Saude Ltda ME, Rua Mario Pederneiras, 00055, APT 103 BLC II, Humaitá, Rio de Janeiro, 22261-020 Brazil; 15 Life Sciences Group, Data for Decisions, LLC, 681 Main Street, Suite 3-37, Waltham, Massachusetts 02451 USA; 16 GSK, 20 Avenue Fleming, 1300 Wavre, Belgium; 17 Meningitis Research Foundation, Newminster House, Baldwin Street, Bristol, BS1 1LT UK; 18 Present Address: Institute of Health Economics and Health Care Management, Helmholtz Center Munich, German Research Center for Environmental Health (GmbH), Ingolstädter Landstr. 1, 85764 Neuherberg, Germany; 19 Present Address: Department of Pharmacy Systems, Outcomes and Policy, College of Pharmacy, University of Illinois at Chicago, Chicago, Illinois USA

**Keywords:** Quality of life, Meningococcal disease, Neisseria meningitidis, DELPHI

## Abstract

**Background:**

Value assessment of vaccination programs against serogroup B invasive meningococcal disease (IMD) is on the agenda of public health authorities. Current evidence on the burden due to IMD is unfit for pinning down the nature and magnitude of the full social and economic costs of IMD for two reasons. First, the concepts and components that need to be studied are not agreed, and second, measures of the concepts that have been studied are weak and inconsistent. Thus, the economic evaluation of the available serogroup B meningococcal (MenB) vaccines is difficult. The aims of this DELPHI study are to: (1) agree on the concepts and components determining the burden of MenB diseases that need to be studied; and (2) seek consensus on appropriate methods and study designs to measure quality of life (QoL) associated with MenB induced long-term sequelae in future studies.

**Methods:**

We designed a DELPHI questionnaire based on the findings of a recent systematic review on the QoL associated with IMD-induced long-term sequelae, and iteratively interviewed a panel of international experts, including physicians, health economists, and patient representatives. Experts were provided with a controlled feedback based on the results of the previous round.

**Results:**

Experts reached consensus on all questions after two DELPHI rounds. Major gaps in the literature relate (i) to the classification of sequelae, which allows differentiation of severity levels, (ii) to the choice of QoL measures, and (iii) to appropriate data sources to examine long-term changes and deficits in patients’ QoL.

**Conclusions:**

Better conceptualisation of the structure of IMD-specific sequelae and of how their diverse forms of severity might impact the QoL of survivors of IMD as well as their family network and care-providers is needed to generate relevant, reliable and generalisable data on QoL in the future. The results of this DELPHI panel provide useful guidance on how to choose the study design, target population and appropriate QoL measures for future research and hence, help promote the appropriateness and consistency in study methodology and sample characteristics.

**Electronic supplementary material:**

The online version of this article (10.1186/s12955-019-1159-0) contains supplementary material, which is available to authorized users.

## Focus on the patient

### What is the context?

- Invasive meningococcal disease (IMD) has the highest incidence rate in young children. In the short term, IMD causes meningitis and/or sepsis. In the long term, IMD survivors can suffer from sequelae of various types and severity levels.

- Current available evidence does not fully capture the burden of long-term sequelae in IMD survivors and their societal consequences, mainly due to gaps and weaknesses in methods of assessment.

### What is new?

- A DELPHI questionnaire was designed and submitted to a board of international experts, including medical doctors, health economists and patient representatives.

- The experts reached consensus on optimal and recommended sequelae classification, study sample characteristics, study design, and quality of life measurement instruments and indicators.

### What is the impact?

- The experts’ recommendations should help harmonise methodologies to assess the quality of life of IMD survivors (as well as their close family members and carers) with long-term sequelae in future studies so that more consistent data can be meaningfully compared, combined, and utilised.

- High-quality and relevant data are a prerequisite to support evidence-based public health decision making.

## Background

Invasive meningococcal disease (IMD) potentially leads after an acute phase to serious long-term sequelae and complications and possibly premature death, thus imposing a high burden onto the patients and their carers [1–3]. The disease is caused by the bacterium *Neisseria meningitidis* and predominantly affects infants and young children [4, 5]. Main clinical presentations of IMD are sepsis and/or meningitis. Six serogroups A, B, C, W, X and Y primarily cause IMD [5, 6]. Although IMD occurs globally, the distribution of serogroups varies across regions. While serogroups B, C and Y are the leading cause of IMD in Europe and other regions of higher-income, such as North America, serogroups A, C, W and X are dominant in the meningitis belt of sub-Saharan Africa. However, serogroup W is now also a leading cause of IMD in some European countries such as the United Kingdom or the Netherlands [7–11].

The availability of vaccines and the implementation of universal mass vaccination (UMV) programs for children decreased the incidence of serogroup C in Europe [4, 11]. Decision-making processes on whether to recommend serogroup B meningococcal (MenB) UMV in European countries are concluded or ongoing [12]. Of the reported IMD cases in Europe in 2016, the majority (54%) was attributable to serogroup B, whereas C accounted for 16%. While serogroup B is responsible for most cases of IMD, especially in children under the age of five, serogroups C, Y and W are predominantly responsible for IMD cases in older age groups [4]. Of note, the clinical presentation of IMD caused by serogroup B is not significantly different from that of other serogroups [13, 14]. Further, associations between both the chance of occurrence and severity of sequelae and serogroups are inconclusive due to low case numbers [13, 15, 16].

Although the incidence of MenB-related IMD is relatively low, the disease has high social and economic costs [17, 18]. However, current evidence on the disease burden does a poor job of pinning down the nature and magnitude of the full costs of IMD for two reasons. First, the concepts and components that need to be studied are not agreed upon, and second, measures of the concepts that have been studied are weak and inconsistent. Hence, a proper translation of the burden on an outcome measure that can be applied for economic evaluation is lacking.

Olbrich et al. (2018) argue that the quality of life (QoL) loss caused by IMD cannot precisely be quantified and assessed since published studies are highly heterogeneous regarding the study design and QoL measurement. This is due to a lack of QoL measurements which differentiate between survivors of IMD with and without sequelae, or QoL measurements specific to types and severity levels of sequelae associated with IMD [16]. Gasparini et al. highlight the importance of accurately estimating the incidence of disease and its consequences, i.e. the type and probability of being left with certain sequelae and the corresponding disutility, to correctly account for the decrease in QoL in IMD patients [19].

Moreover, the measurement of QoL in infants and children is methodologically more challenging than in adults, as shown by Herdman et al. (2016) for IMD cases [20]. Additionally, the perception of health and impairments due to illness might change when patients age and reach adulthood, implying further requirements when QoL is measured along the transition to adulthood. This relates especially to the choice of an appropriate valuation technique, the resulting value sets or the necessity to change the instrument [21]. The impact of the various forms of sequelae on the QoL of IMD survivors remains unclear and seems underestimated [8, 16]. Not only does IMD have a direct impact on the patients, it also has indirect consequences for carers, family members and for the society. These wider health effects might also need to be taken into account for economic evaluation [2, 3, 19].

The aims of this DELPHI study were to:

1. agree on the concepts and components determining the burden of MenB diseases that need to be studied; and
2. seek consensus on appropriate methods and study designs to measure the QoL associated with MenB-induced long-term sequelae in future studies.

## Methods

We used a DELPHI panel approach to find consensus on data gaps and the most appropriate study design to measure the QoL associated with IMD sequelae. The purpose of a normative DELPHI approach is to identify and answer questions of what should be and what is desirable in the future, rather than to assess the current, highly imperfect literature [22]. The conventional DELPHI aims at gathering expert opinion and studying its evolution across iterative rounds of discussion, typically gravitating toward a consensus-based group opinion among the participating experts [23].

The iterative process inherent in the DELPHI technique enables the evolution of the experts’ opinion on the specified research topic, where, due to the controlled feedback, experts re-consider and re-assess their perspective by including broader views and additional information provided by the other participating experts [24, 25]. Furthermore, the DELPHI allows the application of both qualitative and quantitative measures. The former strengthens the in-depth understanding of the expert’s answers, whereas the latter provides a ground for statistical analyses of the data to reach a consensus [26, 27].

### The expert panel

In accordance with the expert definitions provided by Keeney et al. (2001), we invited specialists with experience in measuring QoL, especially that associated with meningococcal long-term sequelae [28]. The expert panel comprised epidemiologists, clinicians, paediatricians, psychologists, patient representatives as well as health economists. Each of the experts has profound experience with study designs, data collection, or clinical experience in the treatment of IMD or in the measurement of QoL, especially in children and adolescents. Additionally, we selected experts to cover the patient’s perspective as well as clinical aspects specific to IMD. Experts were from the UK, USA, Germany, Australia, the Netherlands, Canada, Brazil and Norway in order to cover the international interest to patients, public health authorities and clinicians. Ultimately, 16 experts participated in the DELPHI panel ensuring a broad and appropriate pooling of expert opinions [25, 27].

### The DELPHI process

The first DELPHI round was informed by a systematic literature review. The initial questionnaire provided to experts covered the relevant gaps identified in the recent review by Olbrich et al. [16] and comprised 11 questions (Additional file 1). Of those, five questions were formulated as choice tasks, where the experts had to choose one of the pre-defined items. Four ranking tasks asked the respondents to rank *n* given items numerically, where 1 represents the most important and *n* the least important option. Hence, increasing rank numbers correspond to less preferred answers [29]. Two qualitative questions were included to further explore the experts’ opinions. The choice and ranking tasks gave experts the opportunity to explain their answer or to supplement the list of items within each task.

Along with the initial questionnaire, experts received in March 2017 instructions, a glossary, and a detailed introduction providing a summary of the results from the systematic literature review to ensure proper understanding of the questionnaire. The experts answered the first round questionnaire individually (via e-mail) and anonymously not knowing of the other experts, which Dalkey et al. (1962) recommend as a strategy to minimise group-based interactions between experts [23].

The second round of the DELPHI panel was held in a presence meeting in March 2017 enabling the provision of a summary of the first-round responses with a subsequent discussion giving each expert the opportunity to voice or revise his/her previous answers. Subsequently, an adapted questionnaire with eight questions was given to the experts. For the second round, the two qualitative questions 4 and 11 were dropped for parsimony, since potential answers were collected within the first round or recorded and debated during the discussion. We further dropped question 6, which was unanimously answered during the first round and, hence, a consensus on that specific question was already reached. This second questionnaire also provided feedback from the first round. For the choice tasks the answer distribution was given. Regarding the ranking tasks, experts were provided with the group ranking derived from the rank sum of each item per question.

### Analysis, convergence and consensus

All closed questions had to be either ranked or selected by the experts. Descriptive analyses of the results of the ranking and choice tasks were undertaken. We aggregated the individual ranks into a group ranking by calculating the rank sums; since lower numbers represent a better rank, the items with the lowest rank sum will be considered as the preferred options. The minimum and maximum rank, the mean rank, and the mode of each item were analysed (not reported). Additionally, we evaluated the results from the choice tasks with the help of histograms inspecting the frequency distribution of the items for each question.

Convergence was measured using Kendall’s W, a non-parametric test assessing the level of agreement in an expert group. The test statistic ranges from 0 to 1, where 0 means no agreement and 1 represents full agreement among the experts [30]. The convergence in the choice tasks was assessed by comparing the share of experts selecting a given answer category across rounds. Hence, if the experts’ opinion converges towards a mutually accepted option, the share of experts choosing this particular option should increase while the shares for the remaining items decrease.

A consensus was defined differently for rank and choice questions. In terms of the choice tasks, a consensus was reached when 2/3 of the experts agreed on addressing an issue in a certain way and no veto was raised [31]. A veto could be raised due to ethical, legal, or methodological concerns. In the ranking exercises, we considered the top 3 items per question as the consensus solution, if the 3 corresponding items remained constant across the two rounds. Further, we required Kendall’s W to increase from the first to the second round, implying an increased agreement among the raters [30]. In terms of Kendall’s W, the level of consensus was defined as strong for *W* > = 0.7, moderate for *W* = 0.5 and weak for *W* < 0.3 [29].

## Results

We present key results of the two DELPHI rounds conducted in this study from 5 questions. The first-round questionnaire can be accessed in the Additional file 1.

We achieved an expert consensus on all ranking and choice tasks. From the first to the second round of ranking tasks we observed Kendall’s W to be monotonically increasing (Tables 2, 3 and 4), resulting in moderate to strong levels of agreement after the second round. For all but one of the choice tasks a 2/3 majority was achieved after the second round.

### Question 1 – conceptualisation of sequelae

Table 1 tabulates responses to question 1. For the three tier options presented, the first-round votes showed a slight preference for tier three (with 8 votes) compared to tier two with 6 votes, and tier three with 2 votes. At the end of the second round, 13 out of 14 experts voted for tier three indicating an agreement level of almost 93%. During the process, the experts’ comments emphasized the importance of

- a conceptual model of sequelae, with
  - ○ a detailed classification of sequelae,
  - ○ allowing to distinguish the severity of sequelae,
  - ○ reflecting the impact of multiple simultaneous sequelae
- putting in place the processes for measuring sequelae.

**Table 1:** First and second round responses to Question 1

Tier	Frequency	Frequency
	1st round	2nd round
3rd tier	8	13
2nd tier	6	1
1st tier	2	0

Experts also stated that the sought-after concept of sequelae must be meaningfully measurable and therefore requires a sufficiently large number of patients for each category.

### Question 2 – target population for QoL measurement

The results of this ranking task showed good ability to distinguish among the options (Tables 2 and 3). Assuming that all options were equally important for the disease burden assessment, the rank sums of all options would be equally high. However, the difference in the rank sums between the target groups being deemed the most and least important by the experts for a full disease burden assessment ranged from 50 to 62 points in the two younger age groups and from 65 to 72 points in the adult group. Thus, indicating that the experts had a clear preference order over the importance of the individual target groups. The first-round votes showed a strong preference, in both minor as well as severe sequelae and in all age groups, for patients with an IMD history. Further, the closest carer (i.e. parents, spouse or siblings) were important when assessing the impact of IMD on QoL. This pattern was confirmed in the second round. The preference for the response options “patient”, “parents”, “siblings” and “spouse” got stronger, as the rank sums decreased. The experts agreed that besides measuring the QoL losses in patients, negative spillover effects onto the patients’ parents and siblings should also be considered mainly for the two younger age groups (0–5 and 6–18 year olds), whereas in the adult group (> 18 years) the spouse was more important relative to the other groups for the assessment of QoL losses. This observation is confirmed by the monotonic increase in Kendall’s W indicating a strong level of agreement with W ranging from 0.73–0.77 for the two younger age groups, whereas the agreement remains at a moderate level in the adult age groups (Tables 2 and 3).

**Table 2:** Ranking results from Question 2 – light forms of sequelae

Age group	0–5 years		6–18 years		> 18 years	
Group	Rank sum (group ranking)		Rank sum (group ranking)		Rank sum (group ranking)	
	First round	Second round	First round	Second round	First round	Second round
Patients with an IMD history	22 (1)	15 (1)	16 (1)	14 (1)	16 (1)	14 (1)
Spouse	n/a	n/a	n/a	n/a	46 (2)	37 (2)
Parents	28 (2)	27 (2)	31 (2)	28 (2)	48 (3)	39 (3)
Siblings	50 (3)	43 (3)	51 (3)	43 (3)	63 (4)	55 (4)
Peers (e.g. class mates, friends)	69 (4)	60 (4)	63 (4)	57 (4)	66 (5)	60 (5)
Teachers	71 (5)	66 (5)	69 (5)	68 (5)	88 (6)	78 (6)
Health Care Professionals	72 (6)	71 (6)	78 (6)	72 (6)	88 (6)	79 (7)
Kendall’s W	0.56	0.74	0.63	0.77	0.54	0.60

**Table 3:** Ranking results from Question 2 – severe forms of sequelae

Age group	0–5 years		6–18 years		> 18 years	
Group	Rank sum (group ranking)		Rank sum (group ranking)		Rank sum (group ranking)	
	First round	Second round	First round	Second round	First round	Second round
Patients with an IMD history	22 (1)	14 (1)	17 (1)	14 (1)	16 (1)	14 (1)
Spouse	n/a	n/a	n/a	n/a	42 (2)	37 (2)
Parents	27 (2)	28 (2)	29 (2)	28 (2)	44 (3)	38 (3)
Siblings	49 (3)	42 (3)	49 (3)	42 (3)	62 (4)	55 (4)
Peers (e.g. class mates, friends)	68 (4)	60 (4)	65 (4)	57 (4)	70 (5)	60 (5)
Teachers	72 (5)	64 (5)	67 (5)	66 (5)	86 (6)	79 (6)
Health Care Professionals	73 (6)	71 (6)	78 (6)	72 (6)	88 (6)	81 (7)
Kendall’s W	0.37	0.73	0.39	0.75	0.57	0.64

### Question 7 – viable measures for QoL assessment

The first-round votes resulted in heterogeneous responses and low agreement (Kendall’s *W* < 0.3) on using any of the presented methods to measure the QoL for the two IMD patient age groups (Table 4). However, there was a slight preference for using established measures for both age groups (8–18 years and > 18 years), i.e. existing generic or disease-specific instruments as well as the visual analogue scale. During the second round (Table 4), the preference for these measures became stronger. By contrast, methods of direct preference elicitation, where respondents are asked to attach a value to their health state directly, were ranked lowest independently of the patient’s age, except for those using a visual analogue scale. The agreement level on the ranking of the alternatives reached a moderate level after the second round. During the discussion, experts raised requirements for the questionnaire. It should be cross-country validated, age adequate, and cross-study comparable. Consequently, it was concluded that either of the first to third ranked measures is a sensible choice. Alternatively, developing a disease-specific and age adequate instrument should be considered.

**Table 4:** Ranking results to Question 7

Age group	8–18 years		> 18 years	
Method	Rank sum (group ranking)		Rank sum (group ranking)	
	First round	Second round	First round	Second round
Using an existing generic instrument	33 (1)	23 (1)	45 (1)	22 (1)
Using an existing disease-specific questionnaire	34 (2)	34 (2)	48 (2)	34 (2)
Direct preference elicitation using a Visual Analogue Scale	44 (3)	46 (3)	48 (2)	50 (3)
Using a Discrete Choice Experiment	59 (5)	63 (5)	60 (4)	59 (5)
Direct preference elicitation using the Time trade-off	74 (7)	81 (7)	64 (5)	83 (7)
Direct preference elicitation using the Standard Gamble	70 (6)	75 (6)	65 (6)	75 (6)
Develop a new disease specific questionnaire	49 (4)	50 (4)	66 (7)	56 (4)
Kendall’s W	0.23	0.49	0.07	0.50

### Question 9 – demand for more longitudinal data

**Fig. 1:**
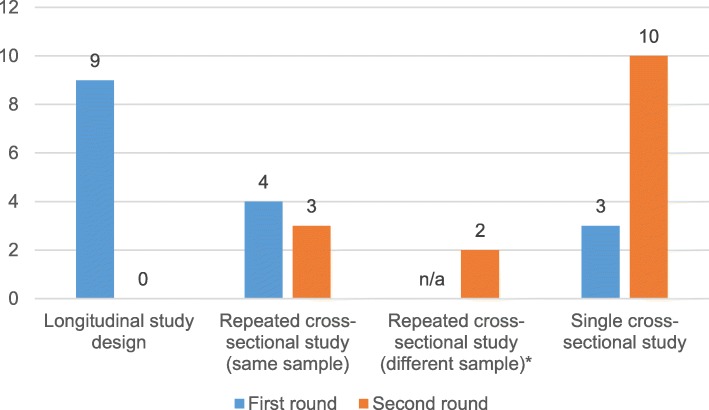
First and second round responses to Question 9. *The distinction between studies using the same or different sample(s) was not considered during the 1st round

Figure 1 summarises the responses given during both rounds. During the first DELPHI round, 9 experts voted for a longitudinal design, whereas 7 experts chose a cross-sectional design. There was also agreement that 2–3 repetitions of the survey were sufficient, and there was almost equal opinion on the follow-up period: 3–20 years with a mode of 5 years. Two experts noted that cross-sectional surveys might be sufficient given that the conduct of long-term longitudinal studies is difficult and costly. Nonetheless, they preferred a longitudinal design. The distinction between studies using the same or different sample(s) for a repeated cross-sectional study was not considered during the 1st round. Given the preference for a longitudinal study design, this option was made available to address this characteristic. The results from the second round show a shift from a longitudinal study to a cross-sectional design, as all participants voted for a cross-sectional design. Finally, the experts agreed that the minimum requirement for a disease burden study would be a single cross-sectional design (67% of experts). They also indicated that a repeated cross-sectional study would be preferred.

### Question 10 – choice of an observational study design

During the first round, 4 experts selected Cohort study, 6 selected Case-control study, and 6 selected Cross-sectional study. It should be noted that the case-control design was defined as a retrospective comparison of health status between patients with a history of IMD (cases) and no such history (controls). Thus, after the first round, a tendency towards pragmatic and less costly study designs was observed. In the meeting prior to the second round, this question was discussed more in depth and in the light of the preceding question 9. In the second round, eight experts voted for a cross-sectional design, while 6 experts preferred a case-control study and 2 experts chose the cohort study design, hence with 50% of the respondents voting for a cross-sectional design no 2/3 majority for a specific observational study design was reached.

The experts reasoned that in a cohort study the evolution of sequelae could be assessed; however, it would be time-consuming and expensive. By comparison, a case-control study as defined above addresses the rarity and long latency of IMD and its corresponding sequelae, allows comparison against healthy controls and covers a variability of patients. Nevertheless, case-control studies are prone to bias. Finally, a cross-sectional study can be supplemented with retrospective data, would also allow to include a control group, is easy to conduct, inexpensive and therefore most practical. No concerns about direction of causality were raised.

## Discussion

IMD is an uncommon, but severe disease. MenC UMV programs in Europe helped decrease the MenC related disease burden. While MenB is still responsible for the majority of IMD cases in Europe, respective UMV programmes are currently implemented less frequently in European countries. Studies evaluating the cost-effectiveness of uncommon but severe diseases such as MenB are challenging and draw on incomplete evidence on the actual disease burden for this particular serogroup [8, 19].

Our results reinforce the need for a more detailed examination of sequelae attributable to IMD and the direct long-term consequences for patients’ QoL as well as the indirect impact onto their parents, siblings or spouse. Experts argued that a detailed classification of sequelae should distinguish both the severity of sequelae and the impact of having multiple simultaneous sequelae. The experts’ perception of an unclear structure of sequelae accords with the findings of a recent literature review on this topic, which reports considerable heterogeneity in prevalence rates and inconsistent sampling strategies [16]. The experts emphasised that a valid operationalisation of the postulated classification is a pre-requisite for a comprehensive QoL measurement. Similarly, Viner et al. (2012) argued that the lack of reliable QoL data prevents the correct assessment of disease burden associated with IMD [8].

On the other hand, the sought-after classification of sequelae should also be pragmatic, since highly stratified patient samples cannot be achieved in future prospective studies due to the small number of patients worldwide as well as the rarity of some sequelae. For example, in 2015 only 1682 prevalent serogroup B IMD cases were reported in countries of the European Union [12].

Generally, the expert consensus on the remaining questions of this DELPHI study suggests pragmatic and goal-driven methods for future studies to overcome the current lack of knowledge, which is impeding rational decisions on the allocation of resources to vaccine development and delivery. The experts acknowledged budget and practical constraints in their answers, and therefore preferred a single cross-sectional study. Further, it was argued that a strategic sampling approach, targeting patients who contracted the disease at different times, as well as the inclusion of a control group might help to examine the evolution of sequelae and to assess QoL losses. However, with respect to the study design we did not achieve a 2/3 majority for a specific design; nonetheless, a tendency towards a retrospective data study with a control group design was observed.

The preference for existing generic and disease-specific QoL measures also reflects pragmatism. However, it should be acknowledged that disease-specific QoL measures are problematic, since they usually do not allow comparisons across different disease areas. They either need to be preference-based or mapped onto a generic preference-based measure in order to be comparable across disease areas or to produce utilities for use in cost-utility or modelling studies, which was desired by the experts. Further, the choice of an appropriate QoL measure is constrained by decision-making bodies in several countries, such as the National Institute for Health and Care Excellence (NICE) in the United Kingdom [32].

Moreover, if the aim is to measure QoL across different age groups, as it is necessary for IMD cases, the assessment of health states along the transition to adulthood is complicated. For this purpose, several established and child-specific QoL measures were discussed. To date, standard paediatric QoL measures, such as the EQ-5D-Y [33] or the Child Health Utility 9D (CHU9D) [34], suffer from at least one of three key limitations. First, validation for the youngest age group is still pending; second, comparability with QoL questionnaires for adults is limited; and third, the scoring mechanisms to translate the children’s answers into a utility score might only be available from adult populations. While the EQ-5D-Y does not offer a child specific value set yet, the EQ-5D-Y broadens the applicable age range (4 to 17 years) and enables the comparison with the instrument for adults by using the same descriptive system. In this sense, the EQ-5D [35] could be applied, as the instruments offer some methodological advantages over other paediatric instruments [21].

Since IMD mainly affects children and adolescents, negative spillover effects on family members and carers must be considered in a comprehensive disease burden study. Our results suggest that, as a practical matter, these primarily need to be considered for the family network and carers, i.e. the parents or siblings of patients ≤18 years. The expert panel agreed that the relevance of the closest relatives and carer, i.e. the parents or the spouse, spans all the included age groups and severity levels. Our conclusion accords with findings from Al-Janabi et al. (2016), who found the most impactful health spillovers in close family members, but with a declining rate with increasing social distance to the patient [2]. Thus, the targeted group in a future IMD-related QoL study should at least consist of patients and their closest relatives and carer, irrespective of the severity of sequelae and age of the patient.

Our DELPHI study of an expert panel with an international composition and multi-disciplinary experience yielded robust results showing a high level of consensus after the second round, reaching a moderate to strong level of agreement on ranking tasks and a majority of 2/3 in choice tasks, except for one question. The structure of our study combined the advantages of anonymity in the first round with the conveniences of a group discussion prior to the second round, where all experts were given the chance to elaborate on their opinion. Given the often not observable or quantifiable differences in the disease outcome between serogroups found in studies, the guidance from this study may help to improve the understanding of the meningococcal disease burden in general, but may also do so by emphasizing MenB to detect potential differences in the disease outcome compared to other serogroups. Several limitations of this study should be acknowledged. Despite providing equal opportunities during the discussion, we cannot rule out that some arguments were prioritised over others. It is inherent to the DELPHI technique that the results will depend on the choice and composition of the expert panel and on how the questions were framed. In this sense, we note that the position of patient representatives was slightly underrepresented numbers-wise. To achieve appropriate framing of the questions, the questionnaire underwent quality control checks within the study group. Finally, the individual expert views and the degree of consensus among them could have changed between the time the DELPHI meeting took place and the time the study was written and reviewed. A long-term follow-up survey might be undertaken in some future study.

## Conclusion

A better conceptualisation of the structure of IMD-specific sequelae and of how their diverse forms of severity might impact the QoL of survivors of IMD as well as their closest relatives and care-providers is needed to generate reliable and generalisable data on QoL in the future. The sought-after conceptualisation builds the basis to overcome the general lack of reliable and relevant measures of the health burden attributable to serogroup B IMD. However, the results of this DELPHI panel provide useful guidance on how to choose the study design, target population and appropriate QoL measures for future research. This will help promote the consistency in study methodology and sample characteristics.

## Additional file

Additional file 1: The first-round DELPHI questionnaire. (DOCX 225 kb)

## Abbreviations

CDC: Centers for Disease Control and Prevention
ECDC: European Centre for Disease Prevention and Control
HRQoL: Health-related quality of life
IMD: Invasive meningococcal disease
MenB: Serogroup B meningococcal
NICE: National Institute for Health and Care Excellence
QoL: Quality of life
UMV: Universal mass vaccination
WHO: World Health Organization
