# Supplementary material for: A DELPHI study on aspects of study design to overcome knowledge gaps on the burden of disease caused by serogroup B invasive meningococcal disease
Source: Health Qual Life Outcomes. 2019 May 22;17:87. doi: 10.1186/s12955-019-1159-0 (PMC6532178; doi:10.1186/s12955-019-1159-0)
Supplement: Supplementary file 1 — The first-round DELPHI questionnaire. (DOCX 225 kb) [file 12955_2019_1159_MOESM1_ESM.docx]

**Online supplement**

**Question 1**

**The literature review identified the following structure of sequelae:**

**
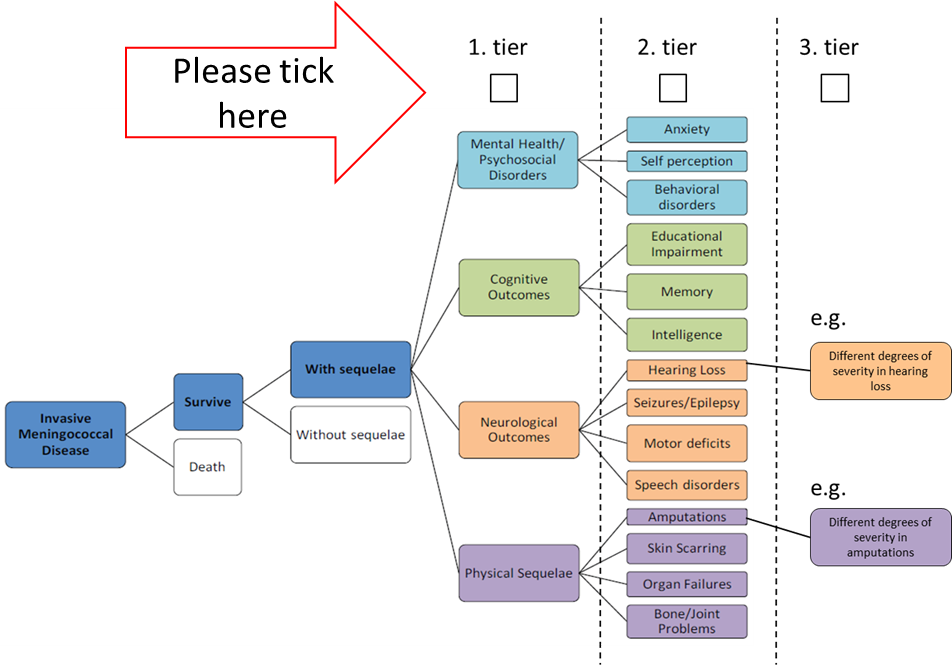
**

**What is the appropriate level of detail for complications you would include in the planned primary data collection study? Please indicate your answer by ticking the box for the corresponding tier in the figure given above and explain your answer. If you wish to add sequelae to the list, do so by using the free text box below. On the contrary, if you consider sequelae from the chosen tier as being not relevant, please cross them out in the figure above. And again, please explain your answer in the free text box below.**

|  |
| --- |

**Question 2**

**Whose HRQoL should be assessed in order to capture the full burden of IMD given that spillover effects might need to be anticipated for parents, siblings, teachers, etc.? If you would like to complement the list please use the table for “others”, state the group you have in mind and rank all of the groups according to your preference.**

**Please consider here light forms of sequelae, which do impair the patient’s life but do not require special care. Please rank the groups for the potential target population for each of the three age groups (3 scenarios).**

| **Age group** | **Rank (0-5 years)** | **Rank (6-18 years)** | **Rank**  **(>18 years)** |
| --- | --- | --- | --- |
| **Group** |  |  |  |
| Patients with an IMD history |  |  |  |
| Parents |  |  |  |
| Teachers |  |  |  |
| Spouse |  |  |  |
| Health care professionals |  |  |  |
| Siblings |  |  |  |
| Peers (e.g. class mates, friends) |  |  |  |
| **Others:** |  |  |  |
|  |  |  |  |
|  |  |  |  |

**Please consider here severe forms of sequelae, which do heavily impair the patient’s life and require special care. Please rank the groups for the potential target population for each of the three age groups (3 scenarios).**

| **Age group** | **Rank (0-5 years)** | **Rank (6-18 years)** | **Rank (>18 years)** |
| --- | --- | --- | --- |
| **Group** |  |  |  |
| Patients with an IMD history |  |  |  |
| Parents |  |  |  |
| Teachers |  |  |  |
| Spouse |  |  |  |
| Health care professionals |  |  |  |
| Siblings |  |  |  |
| Peers (e.g. class mates, friends) |  |  |  |
| **Others:** |  |  |  |
|  |  |  |  |
|  |  |  |  |

**Question 3**

**Preparing a primary data collection study, which population characteristics should be focused on to allow a comprehensive assessment of the burden of IMD and to adjust for confounders in the population? Please rank the characteristics according to your preferences.**

| **Group** | **Rank** |
| --- | --- |
| Cases without sequelae |  |
| Cases with sequelae |  |
| Age |  |
| Gender |  |
| Time since IMD |  |
| Etiology of IMD (MenB vs. MenC) |  |
| Etiology of complications (meningococcal vs. accident) |  |
| Severity of sequelae (e.g. extent of amputation) |  |
| **Others:** |  |
|  |  |
|  |  |

**Question 4**

**Do you expect differences in the burden of disease from IMD induced forms of sequelae compared to the same forms of sequelae induced by other disease? For example, do you expect the severity of hearing loss due to IMD to be comparable to any other form of hearing loss acquired during life time?**

**Please use the free text box below to state the forms of sequelae where you expect these differences.**

|  |
| --- |

**Question 5**

**Please rank the following outcome parameters according to your perceived necessity for future research?**

| **Group** | **Rank** |
| --- | --- |
| Prevalence of overall sequelae |  |
| Incidence of overall sequelae |  |
| Severity levels of overall sequelae |  |
| HRQoL |  |
| Mortality |  |
| **Others:** |  |
|  |  |
|  |  |

**Question 6**

**In general, we rely on patients’ self-reports to assess their respective health states and HRQoL. However, IMD patients might not be able to value their own health states as they are either too young or suffer from cognitive impairments. If that is the case, who do you consider to be the most adequate proxy respondent for the assessment of patient health states?**

|  | Carers (e.g. parents, teachers, spouse) |  | Health Care professionals |
| --- | --- | --- | --- |
|  |  |  |  |
|  | Others |  |  |

**Please, elaborate your answer in the box below – especially when you have chosen “others” as your answer.**

|  |
| --- |

**Question 7**

**In order to find a sensible measure, please rank the following options according to their potential viability in assessing the HRQoL for the two IMD patient age groups stated here.**

| **Method** | **Rank (Children and adolescents 8-18 years)** | **Rank (adults older than 18 years)** |
| --- | --- | --- |
| Using an existing generic instrument |  |  |
| Using an existing disease specific questionnaire |  |  |
| Develop a new disease specific questionnaire |  |  |
| Using a Discrete Choice Experiment |  |  |
| Direct preference elicitation using a Visual Analogue Scale |  |  |
| Direct preference elicitation using the Standard Gamble |  |  |
| Direct preference elicitation using the Time Trade-Off |  |  |

***Note:*** *the underlined terms were defined in an appendix to the original questionnaire.*

**Which existing HRQoL measures or measures under development would you recommend for the measurement of HRQoL in acute patients or patients with a history in IMD? Please state them in the box below.**

|  |
| --- |

**Question 8**

**In the light of low prevalence and incidence of IMD patients a small sample size might need to be anticipated. In this sense, is it viable - in your opinion - to construct special** [**vignette**](#vignette) **describing the health states of interest and to have them valued directly?**

|  | Yes |  | No |  | Undecided |
| --- | --- | --- | --- | --- | --- |

**Please use the box below to give the reasons for your previous answer.**

|  |
| --- |

**Question 9**

**Based on the results of the literature review it has been concluded that there is a need for more robust longitudinal data to clarify the long term effects. Do you agree with that conclusion and what is a reasonable time horizon for this primary data study?**

**Yes**, I agree - a longitudinal study design is necessary to fill the data gaps. Therefore, I recommend a follow-up period of at least _________ years.

**No**, I disagree – a repeated cross-sectional measurement is satisfactory. Therefore, I recommend to conduct at least _________ repeated surveys.

**No**, I disagree – one cross-sectional survey is sufficient to close the existing data gaps.

**Please use the box below to give the reasons for your previous answer. For example, whether you had specific sequelae in mind when you answered the question.**

|  |
| --- |

**Question 10**

**Given your previous answers, what do you think is the most appropriate study design to accommodate the decisions you have made? Please tick the corresponding box.**

|  | Cohort study |  | Case-control study |  | Cross-sectional study |
| --- | --- | --- | --- | --- | --- |

**Please use the box below to elaborate your previous answer. Please, state the reasons you considered (e.g. the patient’s age, specific sequelae) when you answered the question.**

|  |
| --- |

**Question 11**

**In preparation of a primary data collection study, do you have knowledge on potential patient recruitment or data sources, e.g. IMD patient registers, acute treatment centers keeping track of former patients, existing data sets or studies?**

|  |
| --- |
